# A tutorial introduction to adaptive fractal analysis

**DOI:** 10.3389/fphys.2012.00371

**Published:** 2012-09-28

**Authors:** Michael A. Riley, Scott Bonnette, Nikita Kuznetsov, Sebastian Wallot, Jianbo Gao

**Affiliations:** ^1^Department of Psychology, Center for Cognition, Action, and Perception, University of CincinnatiCincinnati, OH, USA; ^2^MINDLab, Aarhus UniversityAarhus, Denmark; ^3^PMB Intelligence, LLCWest Lafayette, IN, USA; ^4^BME, School of Life Sciences and Technology, Xi An Jiao Tong UniversityXian, PR China

**Keywords:** adaptive fractal analysis, time series analysis, fractal physiology, biosignal processing, non-linear analysis

## Abstract

The authors present a tutorial description of adaptive fractal analysis (AFA). AFA utilizes an adaptive detrending algorithm to extract globally smooth trend signals from the data and then analyzes the scaling of the residuals to the fit as a function of the time scale at which the fit is computed. The authors present applications to synthetic mathematical signals to verify the accuracy of AFA and demonstrate the basic steps of the analysis. The authors then present results from applying AFA to time series from a cognitive psychology experiment on repeated estimation of durations of time to illustrate some of the complexities of real-world data. AFA shows promise in dealing with many types of signals, but like any fractal analysis method there are special challenges and considerations to take into account, such as determining the presence of linear scaling regions.

## Introduction

*Adaptive fractal analysis* (AFA; Hu et al., 2009; Gao et al., 2010, 2011) is a relatively new fractal analysis method that may hold promise in dealing with many types of real-world data. In this paper we present a step-by-step tutorial approach to using AFA. We begin by reviewing some basic principles of fractal processes that will be helpful for our presentation of AFA. We then discuss AFA and provide a guide for implementing it. We conclude with an analysis of some synthetic signals and of some real data from an experiment in human cognition.

### Fractal processes

Many physiological and behavioral processes exhibit fractal dynamics. This means the measured patterns of change over time—the behavioral time series—exhibit certain properties, including *self-similarity* and *scaling* (Lebovitch and Shehadeh, 2005). Self-similarity means that the patterns of fluctuations at faster time scales mimics the patterns of fluctuations at slower time scales. Scaling means that measures of the patterns (such as the amount of variability present) depend on the resolution or the time scale at which the measurements have been taken. Many fractal analyses, including AFA, focus explicitly on how a measure of variability scales with the size of a time window over which the measure is calculated. Gao et al. (2007) provided a succinct and comprehensive treatment of various fractal analysis methods.

**Figure 1:**
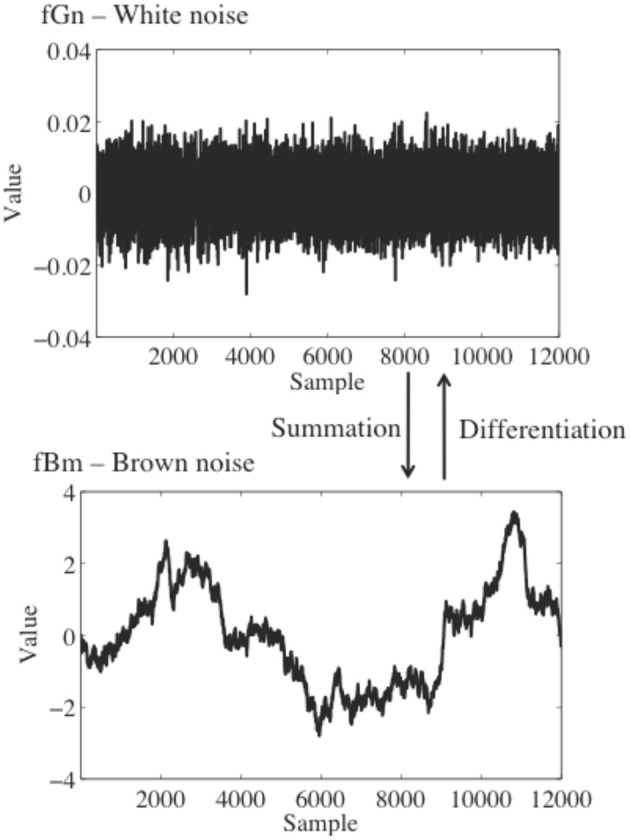
**Top:** A time series of white noise, a fGn process. **Bottom**: a time series of brown noise, a fBm process. A brown noise process can be obtained by successively summing data points in the white noise process.

When conducting fractal analysis of a time series it is important to understand the concepts of *fractional Gaussian noise* (fGn) and *fractional Brownian motion* (fBm), and the differences between the two. fGn is a stationary, long-memory process, whereas fBm is a non-stationary, long-memory process (Mandelbrot and van Ness, 1968; Beran, 1994; Mandelbrot, 1997). Roughly speaking, stationary processes fluctuate by a relatively constant degree around a mean value that remains relatively constant over time, whereas for a non-stationary process the statistical moments of the process (e.g., mean and variance) are time-dependent. “Long-memory” means that the processes exhibit statistical dependencies (correlations) over very long time scales, as opposed to a process for which only adjacent or nearly adjacent data points are correlated with each other. Figure 1 depicts sample time series of fBm and fGn processes.

fGn and fBm are, nominally, dichotomous types of signals. While this is true in an important sense, fGn and fBm are nonetheless related. The increments of a fBm process (created by differencing the signal, i.e., subtracting each value in the time series from the prior value) form a fGn signal [see Eke et al. (2000), for a detailed description of the fGn-fBm dichotomy]. Stated differently, successively summing the data points in a fGn time series will produce a fBm time series. As described below, fGn and fBm require different treatment when using fractal methods to analyze their temporal structure, and the results of a fractal analysis on these two different types of signals will necessarily have different interpretations.

A parameter called the Hurst exponent, *H*, provides a way to quantify the “memory” or serial correlation in a time series. The exact meaning of *H* depends on whether a signal is fGn or fBm. *H* values indicate the correlation structure of a fGn signal, but for a fBm signal the *H* values refer to the correlation structure of the increments obtained by differencing the time series (Cannon et al., 1997). It is therefore necessary to carefully classify a signal as fGn or fBm (or some other kind of signal) before proceeding with fractal analysis of the signal.

With that caveat noted, different *H* values indicate different types of long-memory. Actually, *H* = 0.5 indicates the absence of long-memory (i.e., the process is random—it possesses no memory meaning that data points are uncorrelated with each other) or possesses only short-memory (correlations across very small scales only). This can be considered a null hypothesis of sorts when conducting a fractal analysis; one is often interested in determining whether the data possess some sort of temporal structure rather than being just a truly random, uncorrelated process.

A finding of 0 < H < 0.5 indicates an anti-correlated or *anti-persistent* process for cases of fGn and fBm, respectively. This means that increases in the signal (for fGn) or in the increments of the signal (for fBm) are likely to be followed by decreases (and decreases are likely to be followed by increases)—a negative long-range correlation. In contrast, 0.5 < H < 1 indicates a correlated process for fGn or what is termed a *persistent* process for fBm. In this case, increases in the signal (for fGn) or in the increments of the signal (for fBm) are likely to be followed by further increases, and decreases are likely to be followed by decreases (i.e., a positive long-range correlation). Anti-persistent and persistent processes contain structure that distinguishes them from truly random sequences of data.

To reiterate the point made earlier, and as Eke et al. (2000) carefully explained, an important first step in any type of fractal analysis is to determine the basic type of signal one has measured, i.e., whether the signal is fGn or fBm (see also Cannon et al., 1997). Simply plotting the time series can sometimes help the user make a first-pass determination about whether a pre-processing stage of integrating the data is required. Integration is required only if the data are a stationary, noisy increment process (such as fGn; Figure 1). Integration is not advised if the data are a non-stationary random-walk process (such as fBm; Figure 1). The consequences of this choice are important; *H* estimates can be artificially inflated by integration of a signal which should not be integrated, for example, whereas a lack of integration when it should be performed could suggest the appearance of multiple scaling regions separated by a cross-over point when only one scaling region actually exists (see Delignieìres et al., 2003).

Of course, it is often the case that a plot of the time series cannot be easily classified as an increment or random-walk process based on its appearance alone. Eke et al. (2000) presented a strategy for determining the signal type, termed the signal summation conversion (SSC) method, in the context of a broader approach to analyzing physiological signals that might exhibit fractal dynamics. The method essentially involves comparison of results obtained when the signal is integrated versus not integrated. If *H* values for the non-integrated data approach or exceed a value of 1, then integration of the signal is generally not recommended. *H* values for non-integrated and integrated time series generated by an ideal fBm process should differ by a value of 1; if the difference is considerably greater or less than 1 further scrutiny of the data is required, because in that case the data may not fit within the fBm-fGn framework (Gao et al., 2006; Kuznetsov et al., 2012).

### Adaptive fractal analysis

AFA is similar in some regards to detrended fluctuation analysis (DFA; Peng et al., 1994), and many aspects of AFA will be familiar for readers who already understand DFA. We point out some of these similarities in our presentation of AFA to help those readers, although familiarity with DFA is not required. Because of these similarities, AFA shares many of the same advantages as DFA over other fractal methods, such as the fact that *H* estimated by DFA and AFA do not saturate at 1 as is the case for other methods (Gao et al., 2006).

But despite the similarities between the methods, there are important differences which provide AFA with some advantages over DFA. For example, AFA can deal with arbitrary, strong non-linear trends while DFA cannot (Hu et al., 2009; Gao et al., 2011), AFA has better resolution of fractal scaling behavior for short time series (Gao et al., 2012), AFA has a direct interpretation in terms of spectral energy while DFA does not (Gao et al., 2011), and there is a simple proof of why AFA yields the correct *H* while such a proof is not available for DFA [see Equations 6 and 7 in Gao et al. (2011)].

It is important to note that like many other analyses used to quantify fractal scaling AFA cannot be used independently to assert that a process is or is not a fractal process. Because there are non-fractal processes that can falsely give the appearance of fractal scaling and long-range correlations, it is desirable to use other methods for this purpose (e.g., Wagenmakers et al., 2004; Delignieìres et al., 2005; Farrell et al., 2006; Torre et al., 2007).

**Figure 2:**
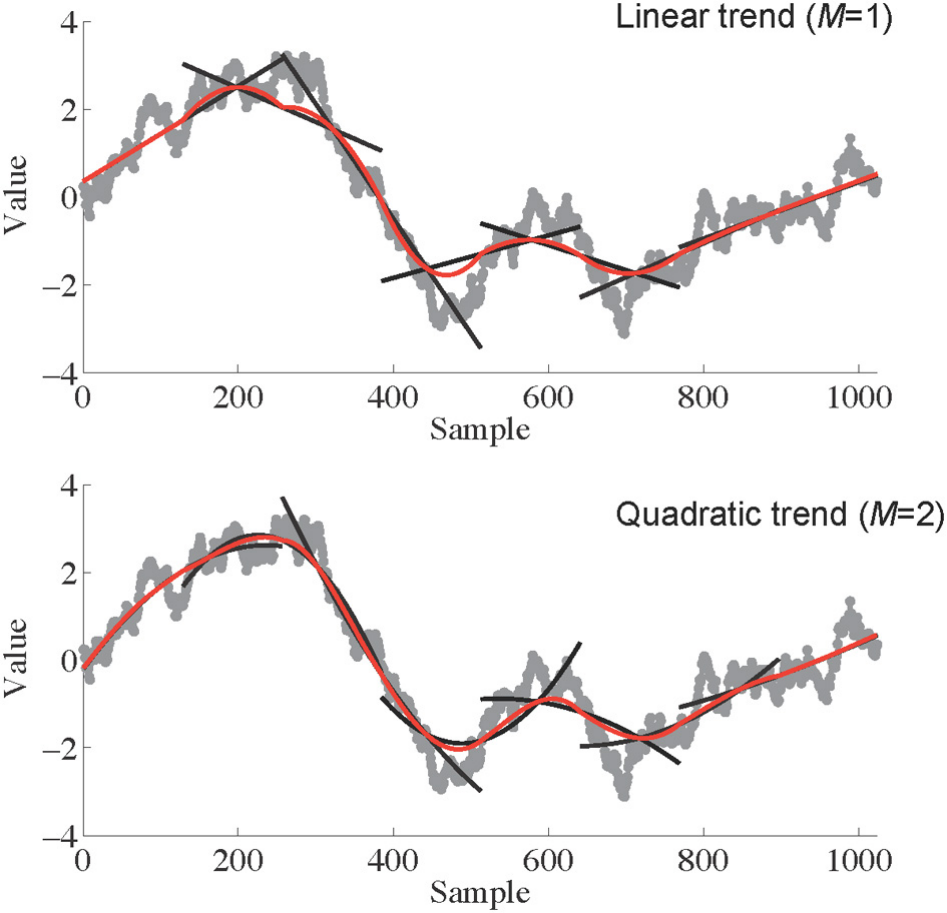
**An illustration of the process of identifying a globally smooth trend signal.** Linear (**Top**; *M* = 1) or polynomial (**Bottom**; *M* = 2) trends are fit to pieces of the signal of length *w* (257 in this case). These fits are shown as black lines superimposed on the original data series (gray curves). The local fits are then stitched together (see Equation 1) to create a smooth global trend signal, depicted in red. Notice that when the end of the series is encountered only half of the data points in that window are used for the trend without smoothing.

The first step in AFA is to identify a globally smooth trend signal that is created by patching together local polynomial fits to the time series. This is one of the primary differences between DFA and AFA; DFA does not involve the creation of this globally smooth trend, and instead relies on discontinuous, piece-wise linear fits. Basically, creating a globally smooth trend signal means that one tries to recreate local features of the data using simple polynomial functions. An example is shown in Figure 2. Small segments of the time series can be approximated reasonably well by adjusting the parameters of a polynomial regression model.

We can now express these ideas in more precise terms [see also Tung et al. (2011), who provided a thorough description of the detrending scheme that forms the basis of AFA]. The goal of this step of the analysis is to create a global trend—a synthetic time series *v*(*i*), *i* = 1, 2,…, *N*, where *N* is the length of the original time series. We denote the original time series as *u*(*i*). Determination of the global trend is achieved by partitioning the original data *u*(*i*) into windows of length *w* = 2*n* + 1, with the windows overlapping by *n* + 1 points. Since setting *w* (a process we describe below) determines the value of *n* [i.e., *n* = (*w*−1)/2], *n* is not a free parameter that must be chosen.

Within each window the best fitting polynomial of order *M* is identified. This is done through standard least-squares regression—the coefficients of the polynomial model are adjusted until the polynomial fits the data with the least amount of residual error. Increasing the order *M* can usually enhance the quality of the fit, but one must be cautious about over-fitting the data. Typically *M* should be 1 or 2—i.e., a linear or quadratic function. The goal is not to fit every squiggle or variation in *u*(*i*) with the polynomial model, but simply to capture any relatively global trends in the data while leaving enough residual variability to analyze further. Presently, there are no validated, objective criteria for selecting *M*, so careful exploration of different *M* values may be required when analyzing a given time series.

The local fits then have to be “stitched” together in such a way that they provide a smooth global fit to the time series. Without this stitching, the local polynomial fits would be disconnected with each other, as is the case for DFA. The stitching and the resulting smooth trend signal thus represents a major distinction between DFA and AFA. The fit to overlapping regions is created by taking a weighted combination of the fits of two adjacent regions to ensure that the concatenation of the local fits is smooth [mathematically, this means that *v*(*i*) is continuous and differentiable], according to

(1) $$y^{\left(c\right)}(l)=w_{1}y^{\left(i\right)}(l+n)+w_{2}y^{\left(i\;+\;1\right)}(l),\;l=1,2,\ldots ,n+1$$

where $w_{1}=\left(1-\frac{l-1}{n}\right)$ and $w_{2}=\frac{l-1}{n}$. According to this scheme, the weights decrease linearly with the distance between the point and the center of the segment. This ensures symmetry and effectively eliminates any jumps or discontinuities around the boundaries of neighboring regions. In fact, the scheme ensures that the fitting is continuous everywhere, is smooth at the non-boundary points, and has the right- and left-derivatives at the boundary. By choosing the parameters of each local fit to maximize the goodness of fit in each case, and then applying Equation 1 to stitch the local fits together, the global fit will be the best (smoothest) fit to the overall time series. Furthermore, this fitting scheme will work with any arbitrary signal without any a priori knowledge of the trends in the data.

The next step is to detrend the data by removing the global trend signal that was just created. We remove the trend because are interested in how the variance of the residuals of the fit—the more fine-grained fluctuations in the original time series *u*(*i*)—scale with *w*, as described below. This type of detrending is very different than simply removing a linear (or higher-order) fit to the original time series prior to data analysis (cf. Di Matteo et al., 2003); the detrending method in AFA (and DFA) is done locally over windows of varying length *w* but not to the entire time series as a whole. The residuals of the fit of the data to the trend signal are identified by subtracting the global trend from the original time series—we compute *u*(*i*) − *v*(*i*). (This is similar to the detrending step performed in DFA, except that as noted for DFA the local linear fits are not smoothly stitched together to create a globally smooth trend signal, but rather are discontinuous with respect to one another.)

These steps that have been described are then repeated for a range of *w* values (i.e., for a range of time scales). Thus, one must choose a minimum and maximum *w*, as well as the size of the time steps (i.e., increases in *w*) used for the analysis. It is perhaps best to begin with the smallest and largest possible *w* values, i.e., *w* = 3 samples and *w* = *N*/2 samples (or *N*/2 + 1 if the time series has an even number of samples) where *N* is the length of the time series. However, as discussed by Cannon et al. (1997), exclusion of some of the smaller and larger window sizes can increase the reliability of *H* estimates. This may be a helpful step when analyzing signals that show a single scaling region over some intermediate range of time scales, and where issues such as measurement noise or insufficient time series length could cause an apparent breakdown of scaling at smaller and larger time scales, respectively. However, one should first ensure that the regions under consideration for exclusion do not themselves contain distinct types of fractal scaling (i.e., that the signal contains multiple scaling regions) to avoid loss of information about the signal. In light of such considerations, we used a *w* range of 3 to (2^9^ + 1 =) 513 samples for the analyses reported here. Any further adjustments to the *w* range can be determined after the next step in the analysis, when one plots log_2_*F*(*w*) as a function of log_2_*w*, as we describe below in our analyses of sample data (and see Kuznetsov et al., 2012). Typically it is sufficient to use a step size of 1, although there may be occasions when a smaller step size is desired to obtain better resolution for identifying linear scaling relations in the plot. In our experience, a step size of less than 0.5 typically does not provide useful new information, but this is an issue that should be explored for each unique data set.

The next step is to examine the relation between the variance of the magnitude of the residuals, *F*(*w*), and the window size, *w*. For a fractal process, the variance of the residuals scales with *w* (i.e., is proportional to *w* raised to the power *H*) according to

(2) $$F(w)={\left[\frac{1}{N}\sum_{i=1}^{N}{\left(u\left(i\right)-v\left(i\right)\right)}^{2}\right]}^{1\text{/2}}\sim w^{H}.$$

**Figure 3:**
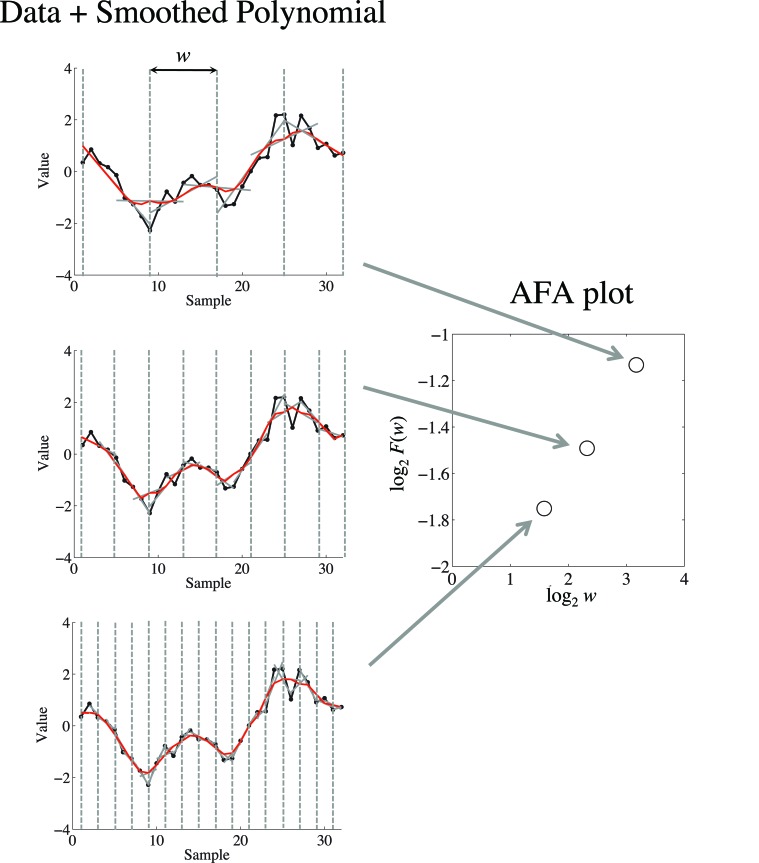
**On the left is depicted a demonstrations of how the fits to different window sizes *w* relate to the AFA plot, shown on the right.** The AFA plot is a plot of log_2_*F*(*w*) (i.e., variance of the residual to the globally smooth trend signal) as a function of log_2_*w* (i.e., time scale or window size). A linear relation in this plot captures fractal scaling, and the slope of the line of best fit provides an estimate of the Hurst exponent *H*. For visual simplicity we only depicted non-overlapping window edges with the dotted gray line, while the analysis uses overlapping windows.

Fractal scaling can be quantified through the slope (obtained using simple linear regression) of a linear relation in a plot of log_2_*F*(*w*) as a function of log_2_*w* (Figure 3). This slope provides an estimate of the Hurst exponent, *H*.

It should be noted that two qualitatively different signals (one fGn, the other fBm) could have the same *H* value. For example, a white noise signal (fGn, so it is integrated prior to analysis) and a brown noise signal (fBm, so it would not be integrated prior to analysis) would both yield *H* = 0.5. Because of this one should use caution performing statistical comparisons of *H* for signals that may differ in regard to being fGn or fBm, and it is partly for this reason that Eke et al. (2000) emphasize the need to report signal classification along with *H* values. For clarity, here we distinguish between *H* for these two processes using the labels *H*_fGn_ and *H*_fBm_.

The above steps constitute the basic process of applying AFA. Often one would perform AFA on each time series in an experimental data set to obtain an *H* value(s) for each, and then submit the set of *H* values to standard statistical analyses (e.g., *t*-test or analysis of variance) to determine if *H* changes across experimental conditions or between groups of subjects. That is, *H* becomes a dependent variable that is analyzed to determine if it changes across levels of some factor.

In the next sections, we apply AFA to known, mathematical fractal processes and then to real-world data obtained from an experiment on human cognition (repeated estimation of the duration of a time interval). The application to known fractal signals demonstrates how AFA is capable of classifying signals in terms of *H*. The application to real-world data reveals the complexities and challenges of using fractal analysis methods to signals that are not idealized fractal processes, like most real signals in the biological, behavioral, and physical sciences. One of these challenges is the matter of deciding how to identify linear scaling regions for AFA (and this challenge applies to other fractal methods, including DFA).

## Applications of AFA

### Application to known fractal processes

Here we present applications of AFA to artificially created time series including some well-studied fractal processes. The advantage of doing so is that we can compare the results of AFA to what should be the “right” answers based on a priori, mathematical knowledge of the artificial time series. Consistent with the goal of this paper to serve as a tutorial for using AFA, we do not mean for this to represent a fully comprehensive test of the method, but rather a straightforward, minimal demonstration that the method correctly identifies these simple “toy” signals. We present results of AFA applied to time series of random, white noise, and two idealized fractal processes known as pink noise and brown noise.

#### Synthetic time series properties

**Figure 4:**
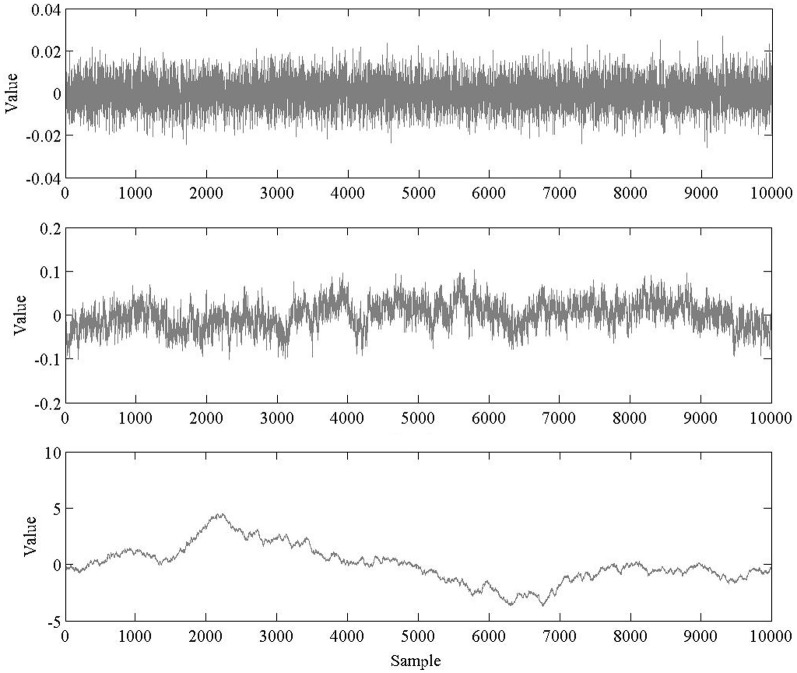
**Sample time series of white (top), pink (middle), and brown (bottom) noise**.

The artificial time series were generated using MATLAB (The MathWorks, Inc.; Natick, MA). Ten time series of length *N* = 10,000 were generated for each of three categories of signals using an inverse Fourier transform (Lennon, 2000): White, pink, and brown noise (see Figure 4). Initially, DFA was used to verify that the synthetic time series we created indeed had the desired mathematical characteristics. The integrated white, integrated pink, and non-integrated brown series were found to have mean (± 1 SD) *H* values of *H*_fGn_ = 0.49 ± 0.01, *H*_fGn_ = 0.97 ± 0.01, and *H*_fBm_ = 0.51 ± 0.01, respectively. The close correspondence between those results and the theoretical values of *H*_fGn_ = 0.5, *H*_fGn_ = 1.0, and *H*_fBm_ = 0.5, respectively, indicates that the simulations produced accurate simulations of fractal processes. Based on our a priori knowledge of the signals, confirmed by visual inspection of stationarity of the time series and these preliminary checks using DFA, only the white and pink noise time series were integrated prior to AFA. The brown noise time series were not integrated.

#### Data reduction and analysis

**Figure 5:**
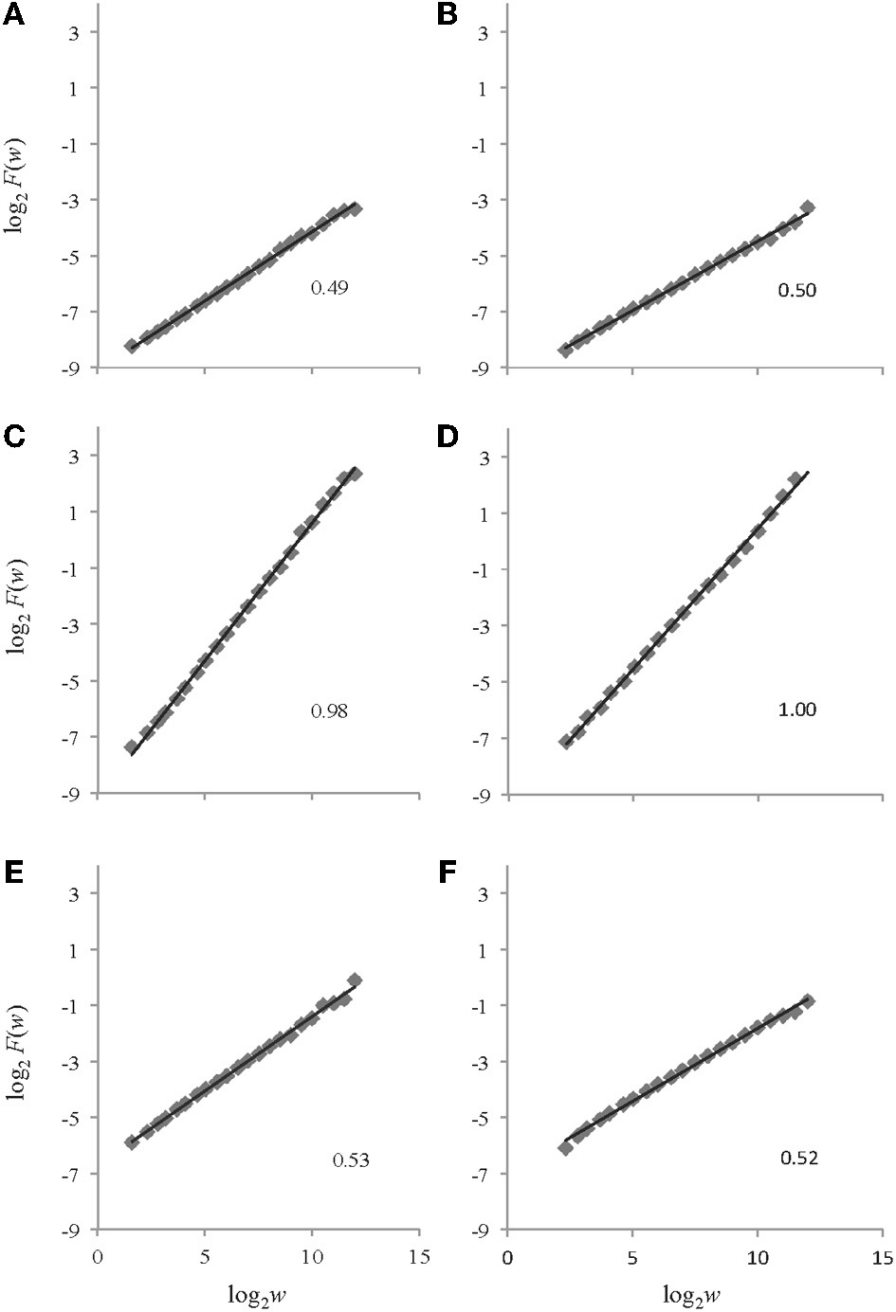
**Example log_2_*F*(*w*) vs. log_2_*w* plots returned by AFA for the time series depicted in Figure 4.** The plots on the left side (panels **A, C,** and **E**) are from AFA using a polynomial order of *M* = 1 while those on the right side (panels **B, D,** and **F**) are from AFA using a polynomial order of *M* = 2. Plots **A** and **B** are for white noise, plots **C** and **D** are for pink noise, and plots **E** and **F** are for brown noise. The respective *H*_fGn_ (**A, B, C,** and **D**) and *H*_fBm_ (**E** and **F**) values are shown for each signal.

The AFA steps described above were implemented on the set of 30 synthetic time series. Parameters of window size *w* = 0.5 and polynomial orders of *M* = 1 and *M* = 2 were chosen for the analyses (AFA was performed once with each polynomial order). Sample AFA plots are shown in Figure 5.

#### Results

For the white noise time series, using polynomial orders of *M* = 1 and *M* = 2, AFA returned mean *H* values of *H*_fGn_ = 0.49 ± 0.01 and *H*_fGn_ = 0.50 ± 0.01, respectively. The pink noise time series were also effectively categorized by AFA in the original time series. A mean *H* value of *H*_fGn_ = 0.98 ± 0.01 was obtained using a polynomial order *M* = 1 and a mean value of *H*_fGn_ = 0.99 ± 0.02 was found using a polynomial order *M* = 2. Lastly, AFA successfully characterized the non-integrated synthetic brown noise time series. Using polynomial orders of *M* = 1 and *M* = 2, AFA returned mean *H*_fBm_ values of 0.51 ± 0.02 and 0.52 ± 0.01, respectively.

#### Discussion

The application of AFA to the synthetic time series indicated that AFA is able to characterize the types of noise with a similar accuracy as DFA. The obtained *H* values corresponded very closely to the theoretically expected values and to the values obtained by DFA (presented earlier). The estimates also exhibited high reliability (low SD values). Changing the polynomial order *M* had very small consequences for these synthetic data; *M* = 2 resulted in slightly better estimates for white and pink noise (and for this polynomial order AFA produced slightly more accurate estimates than did DFA), but slightly worse estimates for brown noise.

### Application to real-world data from a cognitive psychology experiment

We analyzed time series produced by a single participant who repeatedly performed a cognitive task (estimating the duration of a temporal interval) over the course of multiple experimental sessions. The task of repeated temporal estimation is frequently used to study the variability of human time estimation (Delignières and Torre, 2010) and was one of the first reported cases of 1/*f* noise in human cognitive behavior (Gilden et al., 1995).

#### Experimental methods

A single female undergraduate student who gave informed consent participated voluntarily in the study which was approved by the Institutional Review Board at the University of Cincinnati. She was paid $10 per session. The task required the participant to provide repeated estimates of a 1-s time interval. Time estimates were recorded from the presses of the spacebar of a millisecond-accurate keyboard (Apple A1048, Empirisoft). Response times were recorded using the *Psychophysics Toolbox for Matlab* (Brainard, 1997), which recorded the time of each key press during the experiment. We defined one time interval estimate as the time from the beginning of one space bar press to the next one.

At the beginning of each experimental session the participant listened to 20 metronome beats of the 1-s interval to be estimated. The metronome was then turned off, and the participant then immediately began performing the time estimation task. A total of 1050 estimates were produced consecutively in each experimental session, and each session lasted approximately 20 min. There were two experimental conditions that varied with regard to the presence or absence of feedback about the accuracy of the estimates. In the no-feedback condition the participant did not receive any explicit feedback about timing performance. This condition was similar to tasks used previously in continuation tapping experiments (Gilden et al., 1995; Chen et al., 2002; Wagenmakers et al., 2004; Torre and Delignières, 2008). In the feedback condition a computer monitor was used to present feedback specifying the error (in ms) of the most recent estimate on every trial. For example, if the participant hit the space bar 250 ms after 1 s had passed since the previous press, the feedback on the screen would read “250 ms late.” The participant first completed 10 no-feedback trials, one per day on consecutive days, and then completed 10 feedback trials (again one per day on consecutive days). For present purposes we focus on just the first and the last trial in each of the two feedback conditions.

#### Data processing and results

**Figure 6:**
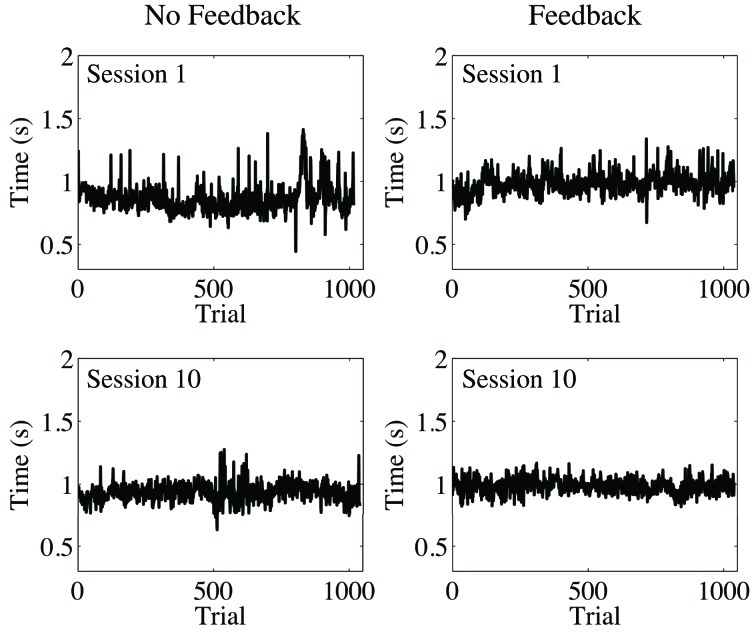
**Trial series of continuous time estimates with and without accuracy feedback after removing observations faster than 300 ms and beyond 3 SD from the mean.** The participant performed the task 10 times in each feedback condition.

We followed the standard procedure in the literature on temporal estimation to remove all observations less than 300 ms and any observations falling beyond 3 SD from the mean. Such values are likely to originate from accidents such as double-tapping the space bar or not initially pressing the bar hard enough, and a significant number of these kinds of outlying values can have detrimental results. From looking at plots of the data processed in this way (Figure 6), it was clear that the time series of temporal estimates were more similar to fGn than fBm (compare to Figure 1)^1^. Therefore, we integrated our data prior to performing AFA. Then, the same basic steps for AFA described previously were again implemented, but with the following additional considerations taken into account. We used *M* = 1 (given that using *M* = 2 did not show consistently better results in our analysis of the sample time series) and log_2_*w* step sizes of 0.5 (because we wanted to enhance the resolution of the AFA plots to facilitate the identification of linear scaling regions).

When dealing with real-world data, if fractal scaling is present it may be limited to a range of time scales (i.e., *w* values). If this is not taken into account, it may lead to inaccuracies in the estimation of *H*. Before estimating *H*, then, it was important to visually inspect the plots of log_2_*F*(*w*) as a function of log_2_*w* to identify regions where linear scaling might be present. If fractal scaling appears limited, it may be necessary to restrict the range of the linear fit to the plot to exclude regions where linear scaling does not occur. Inclusion of regions where fractal scaling is actually absent can lead to inaccuracies and reduce the reliability of *H* estimates (Cannon et al., 1997), and may present an unrealistic picture of the degree to which fractal scaling really is a major feature of the signal being analyzed. In practice, it is desirable to make this process as objective and automated as possible to avoid bias. Elsewhere (Kuznetsov et al., 2012) we have described this issue in more detail, and presented a quantitative procedure designed for this process. For the sake of this tutorial, however, we chose the linear regions visually after inspecting the AFA plots for each trial without the linear fits imposed to examine the possibility of linear scaling.

**Figure 7:**
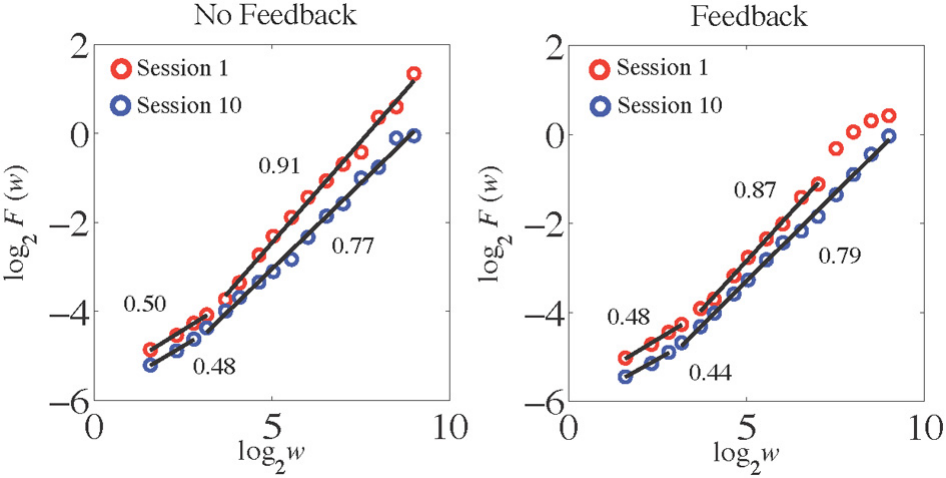
**AFA plots for the time series of time estimates presented in Figure 6.** The *H*_fGn_ values are indicated for each scaling region.

As often occurs with empirical data (as opposed to pure mathematical fractals), some of our time series yielded slightly curved log_2_*F*(*w*) functions (cf. Di Matteo et al., 2003) and had cut-off edge effects especially at larger time scales (*w* > 8 or 256 estimates). Visual inspection of the AFA plots (see Figure 7) suggested two distinct regions of linear scaling, one for low *w* (i.e., fast time scales) and a longer region for higher *w* (i.e., slower time scales), for both feedback conditions and for both the first and last experimental sessions. Such a finding was expected based on previous studies that revealed *H*_fGn_ < 0.5 over the faster scales and *H*_fGn_ > 0.5 at the slower scales (Lemoine et al., 2006; Delignieìres et al., 2008).

In the first experimental session the fast scaling region for the no-feedback condition spanned windows log_2_*w* from 1.58 to 3.17 (in terms of actual number of time estimates this corresponded to a range of 3–9). The *H*_fGn_ value associated with this region was 0.50, indicating the presence of uncorrelated white noise. The slower scaling region for the no-feedback condition had an *H*_fGn_ value of 0.91 (indicating a positive correlation at this scale) and spanned windows log_2_*w* from 3.17 to 9 (13–513 estimates). On the last trial, after a period of practice, the fast scaling region showed a tendency to become slightly anti-correlated but was still very close to white noise (*H*_fGn_ = 0.48) and its length decreased compared to the first session (it now spanned 1.58–2.81 log_2_*w*, or 3–7 estimates). The slow scaling region increased in length (it now spanned from log_2_*w* = 3.17 to 9; 9 to 513 estimates) and became more uncorrelated because its *H*_fGn_ value decreased to 0.77.

A similar pattern of results was found for performance in the feedback condition (see Figure 6, right panel). The fast scaling region during the first session spanned windows from 1.58 to 3.17 log_2_*w* (in terms of actual number of time estimates this corresponded to a range of 3–9) and had a *H*_fGn_ = 0.48, indicating uncorrelated white noise dynamics on this scale. One major difference compared to the no-feedback condition was the shorter length of the slow scaling region in the first session, which now spanned values of log_2_*w* from 3.17 to 8 (9–257 estimates). Similar to the no-feedback condition, the dynamics at this scale exhibited positive correlation as indexed by *H*_fGn_ = 0.87. The breakdown at larger log_2_*w* is likely due to an initial transient evident in the time series plot for this session—for about the first 100 estimates the participant consistently underestimated the 1-s interval, but then began to estimate it more accurately. Because this only happened during one part of the trial, this affected the slowest scaling region of the AFA plot. At trial number 10, similarly to the no-feedback condition, the fast scaling region showed a tendency to become slightly more anti-correlated but was still very close to white noise (*H*_fGn_ = 0.44) and its length decreased compared to the first session (it now spanned 1.58–2.81 log_2_*w*, or 3–7 estimates). The slow scaling region increased in length (it now spanned log_2_*w* = 3.17–9; 9–513 estimates) and became less correlated because its *H*_fGn_ value decreased to 0.79.

#### Discussion

Finite, real-world time series are typically more complex than the ideal simulated noises of mathematics. For example, as was apparent in these time series, experimental data can contain multiple scaling regions. Partly, this may be because experimental data contain both the intrinsic dynamics of the process that generated the signal plus the measurement noise inherent in any recording device. Apart from that, the intrinsic dynamics of real-world signals may have singular events and non-stationarities that if severe enough often can complicate many analyses (including AFA). Because of this it is very important to carefully examine the raw data and the corresponding scaling plots before conducing any quantitative analyses.

With regard to the dynamics of cognitive performance in this temporal estimation task, these results provide preliminary evidence of the presence of practice effects in the continuous time estimation task. Practice led to a decrease in the *H* exponent of the slow scaling region, suggesting that the responses became somewhat more uncorrelated at this scale with practice. Of course our preliminary results have to be interpreted with caution because they are based on single participant and there are individual differences in the slow scaling region *H* values in this task (Torre et al., 2011). The differences between feedback conditions at the fast time scales were not expected because previous literature reported anti-correlated dynamics at this scale (Lemoine et al., 2006; Delignieìres et al., 2008). Feedback clearly resulted in an increased tendency for anti-correlated, corrective dynamics at faster time scales because participants were displayed their performance with regard to the benchmark 1 s time. They appeared to use that information to correct performance on a trial-by trial-basis. In the no-feedback condition, this information was not readily available, which led to essentially random performance at the fast time scales.

## General discussion

We applied AFA to known fractal signals and to real-world data from an experiment in human cognitive psychology that involved the repeated reproduction of a time interval. AFA recovered the *H* values of the known mathematical signals with high accuracy. This was generally true for both *M* = 1 and *M* = 2. The choice of polynomial order did not have a very large effect, although *M* = 2 yielded slightly better results for the white and pink noise signals but slightly worse results for the brown noise signal. Linear scaling was well defined over a single region for these signals.

Application of AFA to the experimental data revealed some of the complexities in applying fractal analyses to real data, particularly the issue of identification of linear scaling regions. We determined the scaling regions visually and then fit lines to them to obtain estimates of *H*. Often this is sufficient, but it is not an objective process and it could be subject to bias in an experiment that involves testing a particular hypothesis or an initial effort to classify a previously unanalyzed type of signal. If visual selection of the scaling region is used, it should be done by multiple observers (so that inter-rater reliability can be computed) who are blind to the experimental conditions and study hypotheses (to avoid bias). In Kuznetsov et al. (2012) we present an objective, quantitative technique based on model-selection methods that could be used to identify scaling regions, but more work remains to be done on this issue.

For the experimental time series we analyzed two linear scaling regions were apparent rather than one. Consistent with previous results using other analysis methods including spectral analysis (Lemoine et al., 2006; Delignieìres et al., 2008), these regions showed distinct slopes. The faster time scale yielded lower *H*_fGn_ and were basically random white noise processes (especially for the no-feedback condition) with a slight tendency toward exhibiting anti-correlated fluctuations. The longer time scale yielded higher *H*_fGn_ values consistent with a correlated process that was close to idealized pink-noise. The presence of feedback had some influence on the structure of the fluctuations of the repeated temporal estimates, as did the practice afforded by performance on consecutive experimental sessions. One of these effects was that linear scaling for the slower time scale broke down at larger *w* for the first session in the no-feedback condition, but spanned the entire upper range of *w* for the last session. These results show that AFA may be sensitive to experimental manipulations that affect the temporal structure of data series both with regard to the estimated *H* values and the range of *w* over which fractal scaling occurs.

Besides the issue of identifying linear scaling region, AFA requires several other choices such as the step size for the window size *w*. Typically 0.5 or 1 log_2_*w* are used, with smaller values providing greater resolution in the AFA plot. In principle this choice should have little impact on *H* estimates, and would not seriously impact computation time except perhaps for extremely long time series. It could, however, have a strong impact on the ability to identify linear scaling regions, especially with regard to resolving the existence of linear scaling regions at faster time scales. The choice of polynomial order *M* for the local fits is also important, especially for signals that may have oscillatory or non-linear trends as higher-order polynomials may be more effective at extracting those trends. Typical choices of 1 or 2 seemed to provide about the same accuracy in estimates of *H* for the known signals we analyzed.

Other factors that impact the ability to identify linear scaling include the sampling rate and the trial length, which, respectively, will affect the ability to resolve faster and slower time scales. These are important choices. A very high sampling rate might indicate the appearance of scaling at very fast time scales, but if those time scales are not physically realistic, one should be cautious about interpreting them. Increasing trial length may help reveal or resolve scaling over very long time scales, which may be very important when dealing with apparently non-stationary time series.

Ideally, AFA should be used in conjunction with other methods, and converging results should be sought. But because AFA but has several advantages over similar methods such as DFA (Gao et al., 2011) the results may not always agree, so care should be taken in interpreting the results. Like all fractal analysis methods, AFA requires careful consideration of signal properties, parameter settings, and interpretation of results, and should not be applied blindly to unfamiliar signals. It is particularly important to plot and carefully inspect the time series and the AFA plots to ensure that the apparent signal properties match with the obtained results. In addition, as we noted previously the appearance of linear scaling regions in an AFA plot is not a definitive test for fractal scaling. When used carefully AFA may provide another useful tool for analyzing signals that may exhibit fractal dynamics.

### Conflict of interest statement

The authors declare that the research was conducted in the absence of any commercial or financial relationships that could be construed as a potential conflict of interest.

## Footnotes

^1^Following suggestions by Cannon et al. (1997) and Eke et al. (2000), we performed spectral analyses on the data to provide a more objective classification of our time series as fGn or fBm. The spectral exponents ranged from 0.48 to 0.75, indicating the signals were consistent with fGn.
